# Prolonged Restraint Stress Increases IL-6, Reduces IL-10, and Causes Persistent Depressive-Like Behavior That Is Reversed by Recombinant IL-10

**DOI:** 10.1371/journal.pone.0058488

**Published:** 2013-03-08

**Authors:** Jeffrey L. Voorhees, Andrew J. Tarr, Eric S. Wohleb, Jonathan P. Godbout, Xiaokui Mo, John F. Sheridan, Timothy D. Eubank, Clay B. Marsh

**Affiliations:** 1 The Dorothy M. Davis Heart and Lung Research Institute, Division of Pulmonary, Allergy, Critical Care, and Sleep Medicine, The Ohio State University, Columbus, Ohio, United States of America; 2 Department of Internal Medicine, The Ohio State University, Columbus, Ohio, United States of America; 3 Division of Oral Biology, The Ohio State University, Columbus, Ohio, United States of America; 4 Institute for Behavioral Medicine Research, The Ohio State University, Columbus, Ohio, United States of America; 5 Department of Molecular Virology, Immunology and Medical Genetics, The Ohio State University, Columbus, Ohio, United States of America; 6 Department of Neuroscience, The Ohio State University, Columbus, Ohio, United States of America; 7 Center for Brain and Spinal Cord Repair, The Ohio State University, Columbus, Ohio, United States of America; 8 Center for Biostatistics, The Ohio State University, Columbus, Ohio, United States of America; University of Minnesota, United States of America

## Abstract

Altered inflammatory cytokine profiles are often observed in individuals suffering from major depression. Recent clinical work reports on elevated IL-6 and decreased IL-10 in depression. Elevated IL-6 has served as a consistent biomarker of depression and IL-10 is proposed to influence depressive behavior through its ability to counterbalance pro-inflammatory cytokine expression. Clinical and animal studies suggest a role for IL-10 in modifying depressive behavior. Murine restraint stress (RST) is regularly employed in the study of behavioral and biological symptoms associated with depressive disorders. While responses to acute RST exposure have been widely characterized, few studies have examined the ongoing and longitudinal effects of extended RST and fewer still have examined the lasting impact during the post-stress period. Consistent with clinical data, we report that a protocol of prolonged murine RST produced altered cytokine profiles similar to those observed in major depressive disorder. Parallel to these changes in circulating cytokines, IL-10 mRNA expression was diminished in the cortex and hippocampus throughout the stress period and following cessation of RST. Moreover, chronic RST promoted depressive-like behavior throughout the 28-day stress period and these depressive-like complications were maintained weeks after cessation of RST. Because of the correlation between IL-10 suppression and depressive behavior and because many successful antidepressant therapies yield increases in IL-10, we examined the effects of IL-10 treatment on RST-induced behavioral changes. Behavioral deficits induced by RST were reversed by exogenous administration of recombinant IL-10. This work provides one of the first reports describing the biological and behavioral impact following prolonged RST and, taken together, this study provides details on the correlation between responses to chronic RST and those seen in depressive disorders.

## Introduction

Major depression currently ranks as the fourth leading cause of disability worldwide [1], [2]. Altered inflammatory cytokine profiles are often observed in depressed individuals [3]–[6]. For example, one of the most consistent biomarkers of depression is elevations in circulating IL-6 [7], [8] and is associated with treatment-resistance [9], [10]. Some animal studies indicate that overexpression of IL-6 promotes depressive-like behavior [11], [12] whereas others are unable to elicit such responses [13], [14]. Recent literature describes concurrent increases in IL-6 and decreases in IL-10 in individuals suffering from major depression [15], [16]. Fluctuations in anti-inflammatory IL-10 are similarly associated with depressive symptoms in humans and are proposed to influence depressive behavior when reduced anti-inflammatory expression is unable to counterbalance the expression of pro-inflammatory cytokines [17], [18]. Additionally, IL-10 plays a role in regulating hypothalamic-pituitary-adrenal (HPA) axis homeostasis by suppressing adrenocorticotropic hormone-induced steroid production and diminished IL-10 expression can affect HPA hyperactivity and glucocorticoid resistance seen in depressed patients [19]–[22]. Importantly, IL-10 treatment ameliorates LPS-induced sickness behavior and depressive symptoms in transgenic mice [23]–[25]. This coincides with both clinical and animal studies indicating that multiple classes of antidepressants elevate IL-10 levels upon successful treatment [26]–[29] and supports the role of IL-10 in affecting depressive behavior.

Rodent restraint stress has been used in modeling human disease for over 85 years [30] and in modeling psychological disease for 35 years [31]. This model is regularly employed in studying behavioral and biological symptoms associated with human depressive disorders [32]–[36]. Although a great deal of work has been done to characterize the biological and immunological events following acute psychological stress exposure, the vast majority of research utilizing RST is conducted over short experimental windows and conveys results obtained at single time points following completion of stress exposure [37]–[41]. Consequently, few studies examine the ongoing and longitudinal effects of extended restraint stress on physiology and behavior. In response, this study was undertaken to provide an examination of biological and behavioral responses to prolonged restraint stress and in the process examine the link between altered peripheral and central cytokine profiles and depressive behavior not only throughout, but also following chronic psychological stress. Here we report that this model of RST (6 hours daily for 28 days) in mice evoked depressive symptoms and cytokine profiles similar to those seen in human depression. This chronic RST resulted in depressive-like complications and altered cytokine expression that persisted for two weeks following stress cessation. Moreover, depressive symptoms induced by RST were rescued by peripheral treatment with recombinant IL-10. Together, this work deepens our understanding of the effects of chronic psychological stress and further supports chronic restraint stress in modeling depressive disorders.

## Methods

### Mice

**Figure 1:**
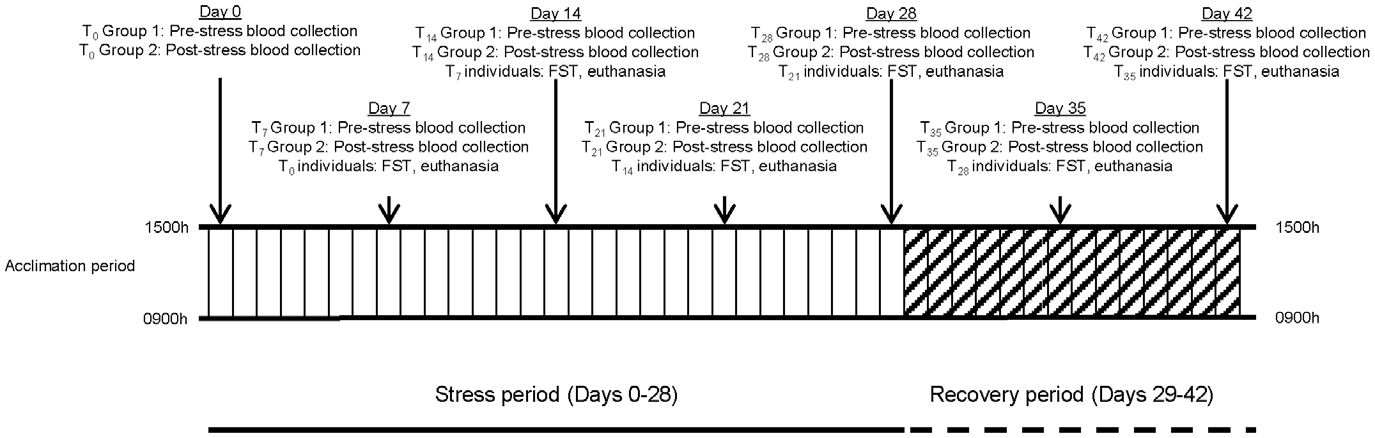
Experimental timeline.

Female C57BL/6J mice age 6–8 weeks were purchased from The Jackson Laboratory (Bar Harbor, ME) and housed in an all-female room in groups of five per cage in an AAALAC-accredited facility on a 12-hour (0600/1800 h) light/dark cycle with *ad libitum* access to standard rodent chow and water. Female mice were selected due to lower incidences of injurious physical interactions. Mice were allowed to acclimate for 7–10 days before exposure to experimental procedures outlined in a protocol approved by The Ohio State University’s Institutional Animal Care and Use Committee and Office of Responsible Research Practices. Mice were handled minimally and humanely throughout the study and no signs of hypothermia or irregular grooming were noted. Mice were humanely sacrificed by CO_2_ asphyxiation. Data included in this report were collected without repeated measurement, sampling, or testing of any individuals (see Figure 1 for graphical representation).

### Experiment 1– Restraint Stress

RST stress experiments were designed and conducted in line with previous reports [42]–[44]. Following the acclimation period, individual cages of animals were randomly assigned to control or RST groups. Beginning the morning of day 0 and concluding on day 28, each animal assigned to the RST group was placed in an individual well-ventilated 50 mL polystyrene tube at 0900 h and returned to its respective cage in a horizontal resting position. At 1500 h RST animals were removed from restraint tubes and allowed to freely move until the next restraint cycle. Control animals were denied access to food and water during the RST period (0900–1500 h) and were otherwise not disturbed. Following the conclusion of the stress period on Day 28, RST and control animals were permitted access to food and water *ad libitum*.

### Experiment 2– Recombinant IL-10 Treatment

Following the acclimation period, individual cages of animals were randomly assigned to control, RST with vehicle treatment, or RST with recombinant murine IL-10 (rIL-10). Beginning on day 1 and concluding on day 21, mice assigned to respective control or RST groups were exposed to restraint stress and treated as described in Experiment 1. Prior to RST on days 14–20, mice were treated by subcutaneous injection with either 50 µL PBS (control and RST group) or 2 µg of rIL-10 (eBiosciences, Sand Diego, CA) in 50 µL PBS.

### Blood Collection

On days 0, 7, 14, 21, 28, 35, and 42, approximately 100 µL of blood was collected from the retro-orbital plexus of experimentally naïve RST and control animals under isoflurane anesthesia (Vedco, St. Joseph, MO) at either 0900 h or 1500 h and blood collection from all animals was completed within five minutes of first handling respective cages in line with previous studies [45]–[47]. Mice were returned to their respective cages following blood collection. Serum was isolated using BD Microtainer Serum Separator tubes (BD, Franklin Lakes, NJ) and stored at −80°C until analysis.

### Forced Swim Test

On Days 7, 14, 21, 28, 35, and 42, approximately 18 hours following completion of the most recent restraint exposure, mice from which blood had been collected one week previously were subjected to a single forced swim test (FST) [48]–[51]. Individual animals were placed in a glass cylinder (43 cm tall, 22 cm in diameter) containing room temperature water (23±1°) filled to a depth of 15 cm. Mice were permitted to move freely for 8 minutes and their movements were recorded. Time to first immobility (defined as 2 consecutive seconds of stationary posture) was recorded as well as total time spent swimming or struggling. Cylinders were cleaned and water changed throughout the swim tests.

### Tissue Collection

On days 7, 14, 21, 28, 35, and 42 mice were euthanized by CO_2_ asphyxiation and immediately weighed. Whole blood was collected by cardiac puncture and serum was isolated using serum separator tubes (BD, Franklin Lakes, NJ) and stored at −80°C until assayed. Spleens, thymuses, and adrenal glands were removed and weighed. The cortex and hippocampus of each individual was isolated and flash frozen in liquid nitrogen before storage at −80°C.

### Corticosterone Measurement

Serum corticosterone levels were assessed using a corticosterone double antibody ^125^I RIA kit (MP Biomedicals, Costa Mesa, CA) according to manufacturer’s instructions. Samples were measured using a Packard Cobra II Auto Gamma counter (Perkin-Elmer Wellesley, MA).

### Serum Cytokine Assay

Serum cytokine levels were measured using a custom BioRad Bioplex assay (Bio-Rad, Hercules, CA) including IL-1β, IL-4, IL-6, IL-10, TNFα, IFNγ with lower detection limits of, respectively, 9.4, 2.1, 0.2, 1.0, 1.2, and 1.4 pg/mL and analyzed using a BioPlex 200 Analysis System (Bio-Rad, Hercules, CA). Serum from RST and control animals from each time point was assayed in duplicate per manufacturer’s instructions. Samples failing to meet internal control standards or below detection limits were omitted. Cytokine levels of control individuals did not vary over time and data presented is relative to control expression levels of each cytokine at each time point.

### RT-PCR

Total RNA was extracted from cortices and hippocampi using standard methods and Tri-reagent (Sigma, St. Louis, MO) according to manufacturer’s recommendations. RNA was assessed for quantity and integrity using a NanoDrop ND-100 Spectrophotometer (NanoDrop, Wilmington, DE). cDNA was produced using Superscript II reverse transcriptase (Invitrogen, Carlsbad, CA). Quantitative PCR was performed using an Applied Biosystems Assay-on-Demand Gene Expression protocol specific for murine samples as previously described [52]. In brief, cDNA was amplified by real-time PCR where target cDNA (IL-1β, IL-4, IL-6, IL-10, TNFα, IFNγ) and reference cDNA (glyceraldehyde-3-phosphate dehydrogenase) were amplified simultaneously using an oligonucleotide probe with a 5′-fluorescent reporter dye (6-FAM) and a 3′-quencher dye (NFQ or TAMRA). Fluorescence was determined on an ABI PRISM 7300 sequence detection system (Applied Biosystems). Data were analyzed using the comparative threshold method and results were expressed as fold-difference.

### Statistics

All data described in this work were collected and analyzed within the structure of a between-subjects design. Data were collected and analyzed without repeated measures and analyzed by ANOVA with two factors (RST and day) except for measurements of corticosterone levels which were analyzed by ANOVA with three factors (RST, day, and AM/PM). The primary comparison was response of RST mice across the stress or non-stress period and comparisons at each specific day and time were further examined. Holm’s method was applied to adjust for multiplicity of the primary outcomes and control the overall family-wise error rate at α = 0.05 [53]. PCR data were subjected to Shapiro-Wilk test using Statistical Analysis Systems (SAS Institute, Inc., Cary, NC) statistical software. Observations greater than three interquartile ranges from the first and third quartile were considered outliers and were excluded in the subsequent analysis [52]. Sample numbers analyzed for each time point are provided as a supplementary table (Fig. S1).

## Results

### Prolonged RST Elevated Corticosterone Levels, Decreased Body Weight, Spleen Weight and Evoked a Sustained Stress Response

**Figure 2:**
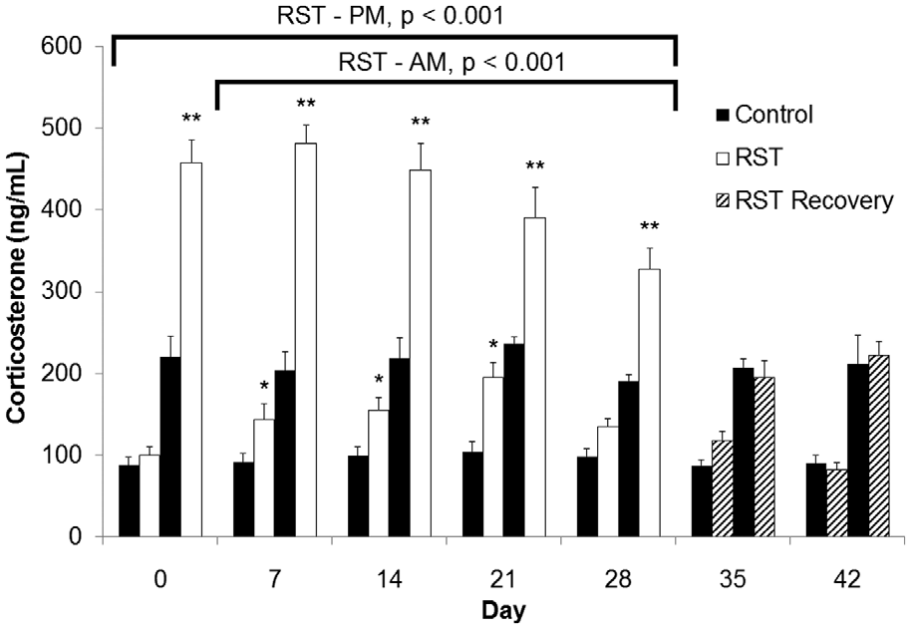
Prolonged RST elevated corticosterone levels throughout the stress period. Corticosterone levels were determined during the stress period (solid bars, Day 0–28) and following stress cessation (hashed bars, Day 29–42). For each data point, n = 5–15 individuals. Data shown is mean +SEM. *p<.05; **p<.0001. Data were collected without repeated sampling of individuals.

**Figure 3:**
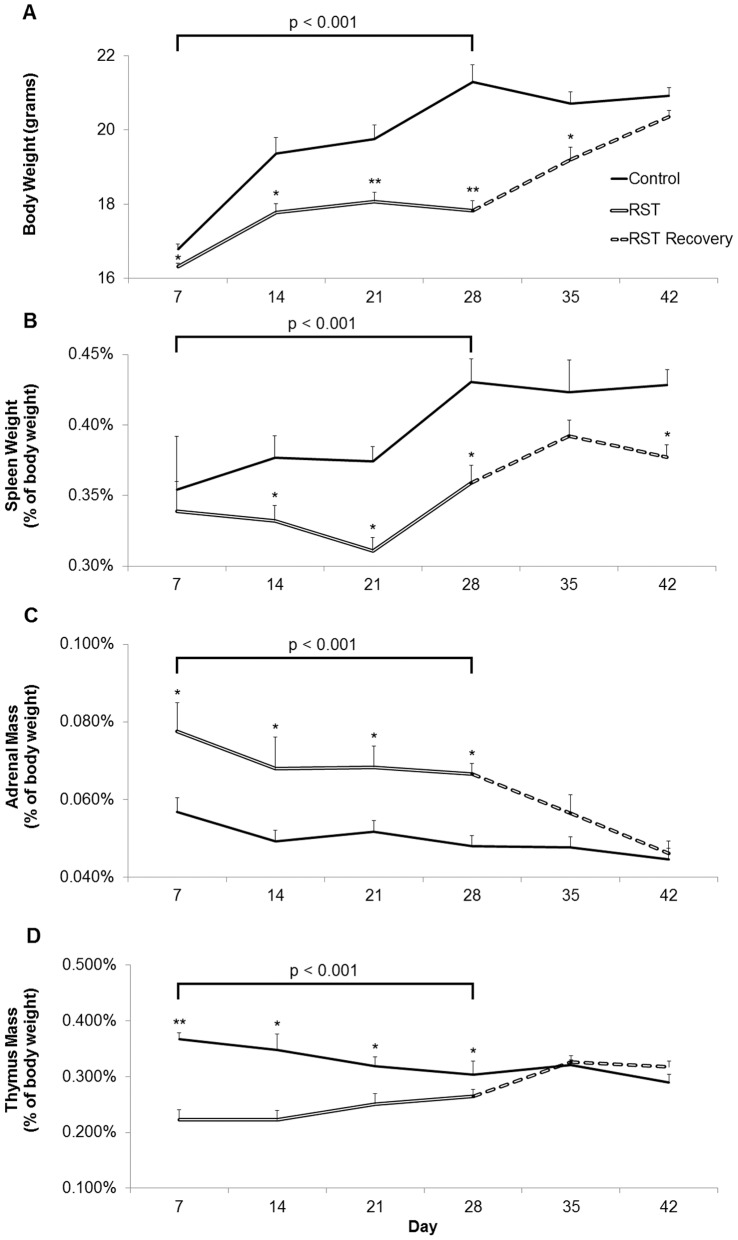
Prolonged RST evoked sustained elevations in stress response. Bodyweight (A), splenic mass (B), adrenal mass (C), and thymic mass (D) were examined during (solid lines, Day 7–28) and following stress cessation (broken lines, Day 29–42). For each data point n = 10 individuals. Data shown is mean +SEM. *p<0.05; **p<.0001. Data were collected without repeated sampling of individuals.

To assess the effects of restraint stress on HPA axis and diurnal corticosterorne rhythm, serum samples were collected from experimentally naïve mice at either 0900 h or 1500 h and assessed for serum corticosterone concentration. Baseline corticosterone measures of control mice were within or below reported ranges [45]–[47]. Prior to initial stress exposure on Day 1, RST and control mice showed no difference in AM baseline corticosterone levels (Fig. 2). After one week, RST increased morning baseline corticosterone levels at 18 hours following the previous stress exposure. Morning corticosterone levels were elevated throughout the stress period (RST 0900 h Day 1–28, p<0.001) and returned to the level of control mice following stress cessation. Corticosterone levels measured immediately following restraint (1500 h) were elevated by RST throughout the stress period (Days 1–28, p<0.001). Corticosterone levels returned to normal afternoon levels one week after the cessation of RST. Body weight was determined over the 42 day time course. RST decreased body weight starting at one week and this reduction was maintained throughout the stress period (Fig. 3A, p<0.001). Following cessation of stress, mice subjected to RST returned to that of control mice. Because stress-induced elevations in glucocorticoid levels induces splenic atrophy in RST mice (Wang et al. 2008), splenic mass was determined during the stress period and following stress cessation. RST decreased spleen weight during the stress period (Day 7–28) compared to controls (Fig. 3B; p<0.001). Spleen proportions increased after the conclusion of RST but remained significantly smaller than control mice two weeks after stress cessation. Previous reports associate restraint stress with increased adrenal and decreased thymus mass (Toth et al. 2008), therefore these parameters were determined during and following the stress period. In accordance with earlier reports, RST increased adrenal gland mass (Fig. 3C; p<0.001) and decreased thymic mass (Fig. 3D; p<0.001) during the stress period (Day 7–28). After a maximum at Day 7, adrenal gland mass decreased throughout the remainder of the stress and post-stress period (Day 29–42). In contrast, thymuses of RST animals recovered rapidly upon completion of the stress protocol (Day 35).

### Restraint Stress Produces a Depressive-like Phenotype both During and following the Stress Period

**Figure 4:**
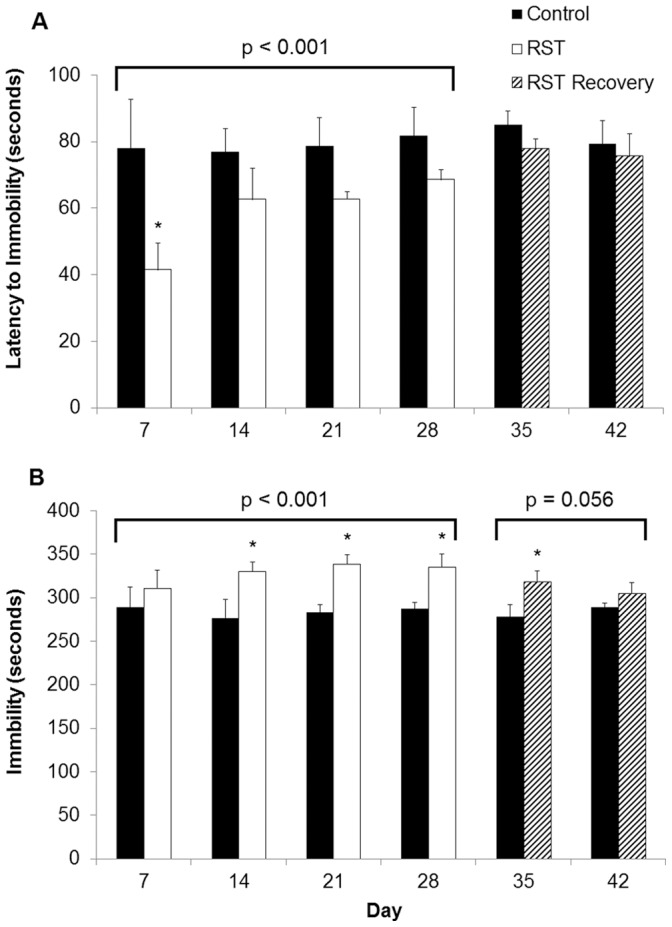
Mice exposed to prolonged RST demonstrated sustained depressive-like behavior. Mice were exposed to FST throughout the experimental period. Time to first immobility (A) as well as total immobility (B) were recorded. For each data point n = 10 individuals. Data shown is mean +SEM. *p<0.05. Data were collected without sampling of individuals.

Previous work associates rodent restraint stress with the development of depressive-like behavior. Time spent immobile [33], [36], [54], [55] and latency to first immobility [55]–[57] are used in quantifying a depressive behavioral phenotype in forced swim tests. RST decreased time to first immobility throughout the stress period (Fig. 4; p<0.001). This was resolved following the completion of RST. Moreover, immobility in the forced swim test was increased throughout the stress period overall (p<0.001) and remained increased seven days following the cessation of RST (Days 35; p<0.05). In addition, RST appears to increase immobility during the post stress period, an effect that was marginally significant (p = 0.056). These data indicate that chronic RST promotes a depressive-like behavior that persists weeks after the cessation of stress.

### Prolonged Restraint Stress Increases Circulating IL-6, Decreased IL-4 and IL-10

**Figure 5:**
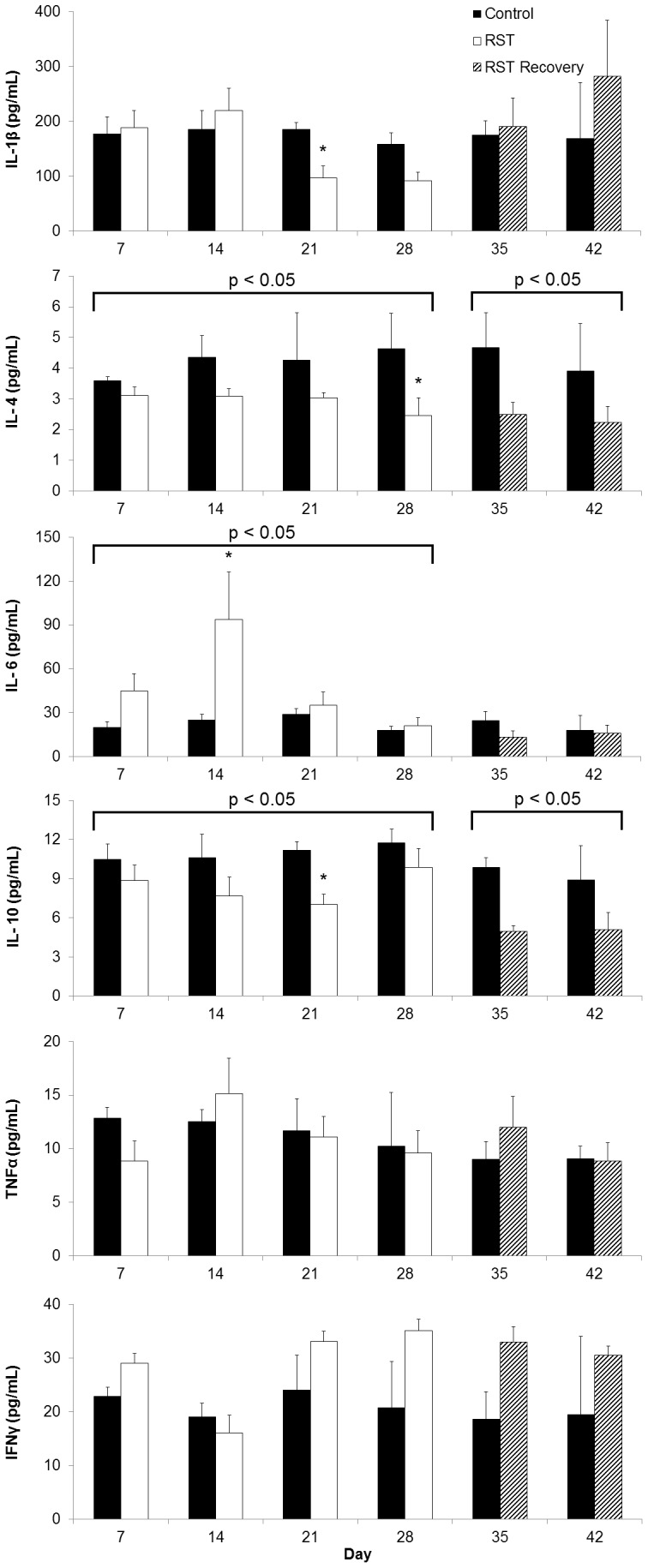
RST elevated circulating IL-6 and decreased IL4 and IL-10. Circulating levels of pro- and anti-inflammatory cytokines were measured throughout the experimental period. For each data point n = 3–9 individuals. Data shown is mean +SEM. *p<0.05. Data were collected without repeated sampling of individuals.

Depressive disorders are associated with altered circulating cytokine profiles [3], [4]. To investigate the role of inflammatory response in depressive-like behavior, serum levels of IL-1β, IL-4, IL-6, IL-10, TNFα, and IFNγ were determined throughout and following the stress period. Control values of cytokines were consistent with previous reports [58]–[60]. RST increased IL-6 (p<0.05) and decreased IL-4 (p<0.05) and IL-10 (p<0.05) in the serum during the stress period (Day 7–28). Moreover, these RST-induced reductions in the serum levels of IL-4 and IL-10 were sustained during the post-stress period (Fig. 5, Day 29–42, p<0.05, and p<0.05, respectively). Despite reduced circulating anti-inflammatory cytokines, inflammatory cytokines including IL-1β, TNFα and IFNγ were not increased in the serum by RST. Data is represented relative to levels of corresponding cytokines of control mice at each time point and cytokines levels of control mice were consistent over time.

### Prolonged Restraint Stress Elicits Suppression of IL-10 in the Cortex and Hippocampus

**Figure 6:**
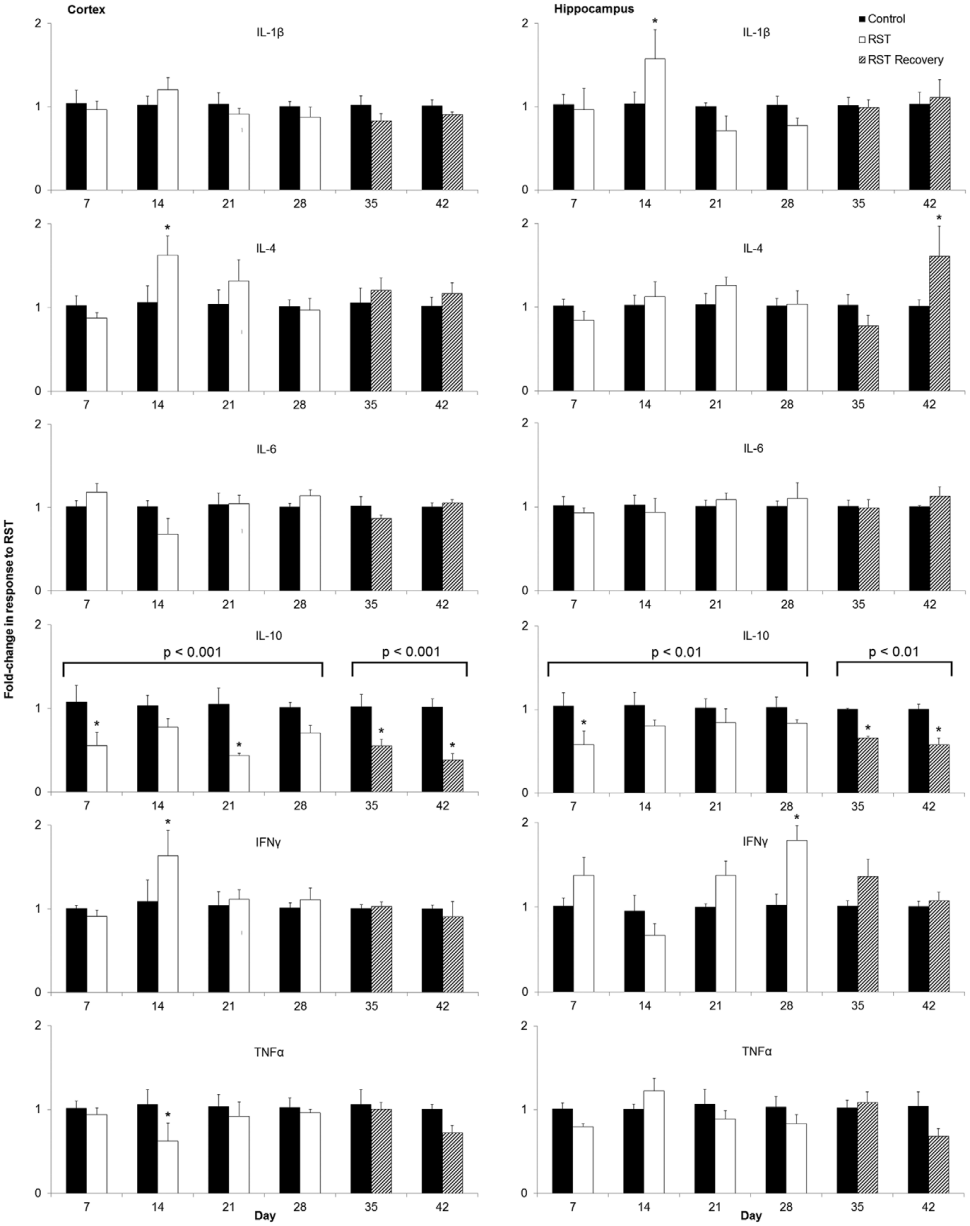
RST decreased expression of IL-10 in the brain. mRNA expression of pro- and anti-inflammatory cytokines were measured in the cortex and hippocampus. For each data point n = 6–10 individuals. Data shown is mean +SEM. *p<0.05.

To determine if the effect of RST on serum cytokines was paralleled in the brain, IL-1β, IL-4, IL-6, IL-10, TNFα, and IFNγ mRNA expression levels were determined in the cortex and hippocampus throughout and following the restraint period. RST decreased mRNA IL-10 expression in the cortex and hippocampus during the stress period (Fig. 6; p<0.001 and p<0.01, respectively; Day 7–28). This RST associated decrease in IL-10 mRNA expression was maintained during the weeks following the cessation of stress (Fig. 6, Day 29–42; p<0.001 and p<0.01, respectively). Similar to serum, pro-inflammatory cytokine mRNA was not increased in the brain during the stress or post-stress periods. Collectively, these data indicate that chronic RST decreases peripheral and central IL-10.

### Recombinant IL-10 Reduced Stress-induced Behavioral Deficits

**Figure 7:**
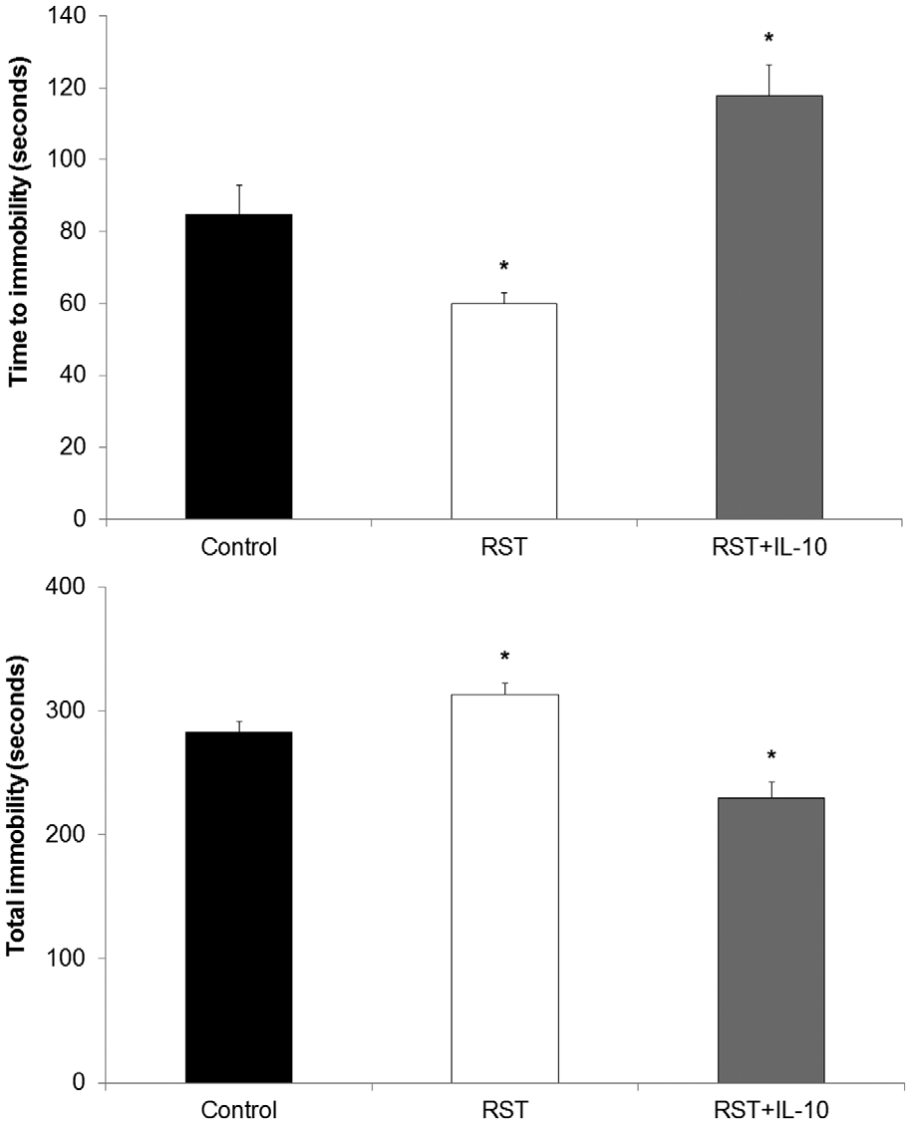
IL-10 treatment exhibits a rescuing effect on restraint stress-induced behavioral deficits. As part of a 21-day RST protocol, mice were treated with vehicle (control and RST mice) or murine IL-10 (IL-10 mice) for seven days immediately preceding FST. For each data point n = 5–15 individuals. Data shown is mean +SEM. *p<0.05.

Because RST decreased IL-10, RST mice were treated with recombinant murine IL-10 or vehicle during the final seven days (days 14–20) of the 21-day restraint stress protocol and depressive-like behavior was determined. As expected, RST decreased time to first immobility (p<0.01) and increased total immobility (p<0.01) compared to controls (Fig. 7). The RST-induced depressive-like behavior was blocked by recombinant IL-10 treatment. For instance, IL-10 treatment extended time to first immobility and decreased total time spent immobile compared to vehicle-treated control mice (p<0.01 and p<0.01, respectively) and RST mice (p<0.01 and p<0.01, respectively).

## Discussion

While a great deal of work has been done to characterize the events immediately following bouts of acute psychological stress, the vast majority of research utilizing RST is conducted over short experimental windows and frequently convey results obtained only at single time points upon completion of stress events [37]–[41]. Consequently, few long-term projects examine the ongoing biological and behavioral effects during extended periods of restraint stress and still fewer studies evaluate responses following stress cessation. For these reasons we extended the experimental timeframe, collecting data throughout both stress and post-stress periods. In proceeding with this investigation, we first characterized markers of stress response. We observed elevated and sustained stress responses throughout the stress period that returned to control levels following stress cessation. While corticosterone levels rise before rapidly returning to pre-stress baseline following acute psychological stress exposure, the model of chronic restraint stress employed here produced baseline AM corticosterone that were elevated even 18 hours following conclusion of the previous stress cycle (Fig. 2; RST - AM). This observation was seen throughout the stress period. These measurements were recorded at a time when corticosterone levels are typically at their lowest point during the diurnal cycle [61], [62] and convey a sustained elevation in minimum daily corticosterone levels throughout the 28-day stress period. Similarly, while PM corticosterone levels showed a maximum response on day 7, corticosterone levels measured immediately upon completion of stress exposure reflected an exaggerated and sustained stress response throughout the 28-day stress period (Fig. 2; RST - PM). Previous work using a 16-hour model of nocturnal restraint demonstrated disruption of diurnal corticosterone patterns following 8, but not 15 days of RST when combined with viral infection [63]. Here we found disruption of daily corticosterone patterns and elevated corticosterone levels that perdured throughout the 28-day stress period, perhaps owing to the sampling of naïve animals rather than repeated sampling of individuals as in other studies. This disruption of regular diurnal rhythm coupled with the inability to recover between RST exposures is indicative of chronic stress. Gross morphological changes have been described as part of an active response to restraint stress and observations of enlarged adrenal glands as well as decreased spleen and thymus proportions throughout the 28-day stress period are in line with previous studies [64]–[66] (Fig. 3). Together this indicates that chronic RST produces sustained stress responses throughout a 28-day RST protocol.

A key finding in this study was that RST caused depressive behavior that extended long after stress cessation. Forced swim tests are used in identifying depressive rodent phenotypes in which diminished total mobility and decreased time to first immobility are viewed as reflecting depressive-like behavior [32]–[34], [48], [54], [57]. Earlier reports demonstrate inconsistent behavioral responses to murine restraint stress: mice exposed to acute bouts of restraint (single exposure, >60 minutes) show, alternately, diminished latency to immobility and increased immobility [67] or no difference in behavior [68]. Additionally, murine restraint studies extending for up to 10 days report results ranging from no difference in depressive-like behavior to species-dependent variability [54]. In this study, application of uniform daily restraint exposure showed that time to first immobility was diminished at the earliest time point (Day 7) and reduced overall during the 28-day RST period (Fig. 4). Further, when the timeframe of examination was extended, mice showed decreased latency to immobility during the stress period which then continued into the period following stress cessation. To our knowledge this is the first report of depressive behavior extending into the weeks following restraint stress exposure and argues for further exploration of the mechanism linking psychological stress and depressive behavior.

The combined role of IL-6 and IL-10 in depression have garnered recent attention with the recognition of human populations suffering from major depression displaying commensurate increases in IL-6 and decreases in IL-10 [15], [16], providing direct biological correlation with this animal model. While elevated IL-6 is itself reported as a biomarker of depression [7], [8], [69], animal studies show the behavioral effects of IL-6 alone to be inconsistent. IL-6 stimulates the murine HPA axis [70] and peripheral administration increases brain tryptophan levels and elevates serotonin metabolism [55], together providing a rationale for depressive behavioral modifications. However, reports have also shown that peripheral IL-6 treatment increases exploratory and locomotive behavior in mice [71] without affecting feeding [72] or reward response [73], and intracerebroventricular IL-6 has alternately resulted in decreased locomotion and reward response [74] or no change in locomotive and social investigatory behavior in rodents [13]. Further, IL-6 KO mice show no difference in depressive-like behavior as measured by tail suspension or forced swim tests [55]. Taken together, this indicates that while increased circulating IL-6 as described here may affect behavior (Fig. 5), altered IL-6 expression cannot alone account for depressive symptoms. The inflammatory status of the cortex and hippocampus are directly implicated in mood disorders and major depression [75]–[77] and while no intracranial elevation in IL-6 was observed here, IL-10 expression was reduced in the cortex and hippocampus of stressed animals both throughout and following the stress period (Fig. 6). Despite suppression of anti-inflammatory IL-10, no corresponding broad increase in pro-inflammatory markers was recorded. This is in line with previous work demonstrating decreased IL-10 expression and increased IL-6/IL-10 ratios in the cortex of rats exposed to an extended unpredictable chronic mild restrain protocol and [78]. This also provides context for the restraint stress model when considering studies showing the inability of 28 days of psychosocial stress to elicit changes in IL-6 or IL-10 mRNA expression in the mouse brain [79].

Whereas acute psychological stress has shown either no effect on circulating IL-10 levels in mice [80] or increased IL-10 levels in rats [81], in line with clinical studies of major depression [15], [16] here chronic restraint stress resulted in diminished circulating IL-10 throughout stress period and for two weeks afterward. These findings describe a systemic peripheral and central suppression of IL-10 both during and following the restraint period. There has been little work describing a role for IL-4 in depressive disorders and decreases in circulating IL-4 observed here may correspond to a more generalized suppression of anti-inflammatory cytokines in response to sustained restraint stress rather than specific features of depressive response. Together, this suggests that depressive behavior may be influenced not only by elevations in pro-inflammatory cytokines, but also independently affected by suppression of anti-inflammatory cytokines. Following from the observations of decreased IL-10 expression and increased measures of depressive-like behavior induced by RST, we found that depressive-like behavior did not develop in mice treated with recombinant murine IL-10 (fig. 7) though further work is require to elucidate the full mechanism. Traditional cytokine-based theories of depression place great emphasis on the role of inflammatory cytokine elevations in depressive illness, though important work suggests that IL-10 itself plays a direct role in affecting depressive behavior. Numerous clinical studies show that multiple classes of effective antidepressants elevate circulating IL-10 from pre-treatment levels upon successful therapy, though no direct mechanism is described [26]–[29], [82]. IL-10 ameliorates LPS-induced sickness behavior in rodents [83] while IL-10-deficient mice show increased fatigue and motor deficits following LPS exposure [84]. Transgenic studies using IL-10 knockout mice show that an absence of IL-10 expression results in constitutively depressive behavior (as measured by FST) and that treatment with recombinant IL-10 can ameliorate these behaviors [24], [25]. Further, IL-10 overexpression decreases measures of depressive behavior in mice in FST assessments and exogenous IL-10 treatment amplifies physical activity and measures of exploratory behavior in wild-type mice [23]–[25]. In this work we observed that chronic restraint stress induced a measure of depressive-like behavior (Fig. 4) and decreased expression of IL-10 in the brain and in the periphery, both during and in the weeks following restraint (Fig. 5 & 5) and further work will include additional examinations of depressive-like behavior such as sucrose-preference tests. FST has been frequently used in assessing antidepressant efficacy, though many recent studies have included it as an independent assessment of depressive behavior [85]–[87]. Whereas IL-6 levels normalized following stress cessation, this pattern of sustained IL-10 suppression corresponded with sustained measures of depressive-like behavior. Moreover, while IL-6 shows inconsistent ability to modify depressive behavior, behavioral deficits brought about by RST were reversible by treatment with recombinant IL-10, thereby highlighting the role of IL-10 in directly affecting behavior. This demonstrates that while depressive disorders may frequently display comorbidity with elevations in pro-inflammatory markers, depressive behavior itself may be attributable to suppression of anti-inflammatory cytokines rather than increases in pro-inflammatory cytokines.

## Supporting Information

Figure S1 **Sample size examined for each data point.**
(TIF)
