# Supplementary figures and images for: Prolonged Restraint Stress Increases IL-6, Reduces IL-10, and Causes Persistent Depressive-Like Behavior That Is Reversed by Recombinant IL-10
Source: PLoS One. 2013 Mar 8;8(3):e58488. doi: 10.1371/journal.pone.0058488 (PMC3592793; doi:10.1371/journal.pone.0058488)

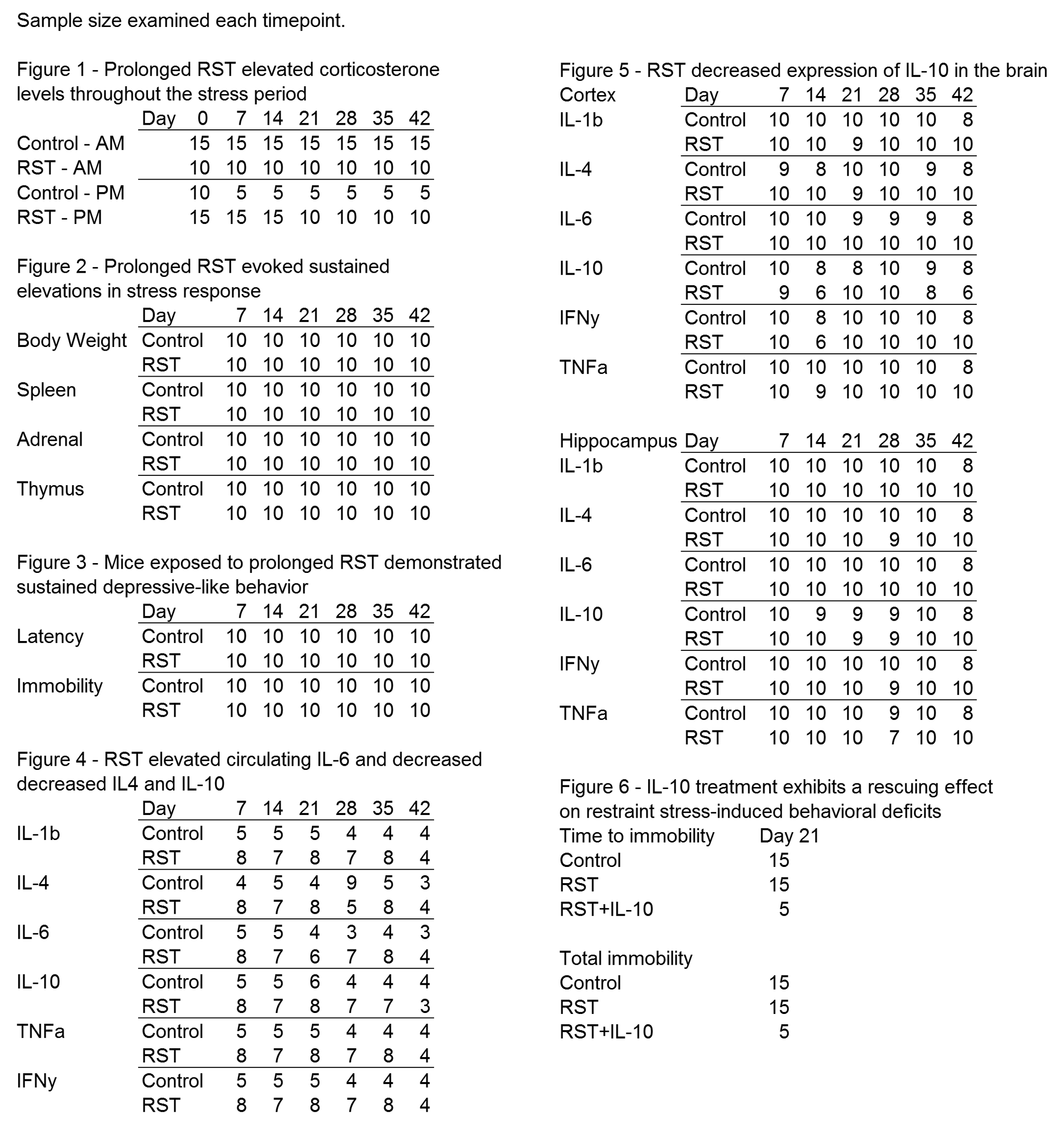

Supplement: Figure S1 — Sample size examined for each data point. (TIF) [file pone.0058488.s001.tif]
